# Omega-3 and -6 fatty acid plasma levels are not associated with liver cirrhosis-associated systemic inflammation

**DOI:** 10.1371/journal.pone.0211537

**Published:** 2019-01-31

**Authors:** Katharina Maria Schwarzkopf, Alexander Queck, Dominique Thomas, Carlo Angioni, Chengcong Cai, Ylva Freygang, Sabrina Rüschenbaum, Gerd Geisslinger, Stefan Zeuzem, Christoph Welsch, Christian Markus Lange

**Affiliations:** 1 Department of Internal Medicine 1, Goethe-University Hospital Frankfurt, Frankfurt, Germany; 2 Institute of Clinical Pharmacology, Goethe-University Frankfurt, Frankfurt, Germany; 3 Fraunhofer Institute for Molecular Biology and Applied Ecology IME, Project group Translational Medicine & Pharmacology TMP, Frankfurt, Germany; University of Illinois, UNITED STATES

## Abstract

**Background:**

Liver cirrhosis is associated with profound immunodysfunction, i.e. a parallel presence of chronic systemic inflammation and immunosuppression, which can result in acute-on-chronic liver failure (ACLF). Omega-3 fatty acids are precursors of pro-resolving mediators and support the resolution of inflammation.

**Objective:**

The aim of this study was to determine plasma levels of omega-3 fatty acids in patients with liver cirrhosis and ACLF.

**Methods:**

Patients with liver cirrhosis with and without ACLF were enrolled in a prospective cohort study and analyzed post-hoc for the present sub-study. Clinical data and biomaterials were collected at baseline and at day 7, 28 and after 3 months of follow-up. Plasma concentrations of arachidonic acid (ARA) and docosahexaenoic acid (DHA), which represent key omega-6 and -3 fatty acids, respectively, were quantified and associated with markers of systemic inflammation and severity of liver cirrhosis.

**Results:**

A total of 117 patients were included in the present analyses. Of those, 26 (22.2%), 51 (43.6%) and 40 (34.2%) patients had compensated or decompensated liver cirrhosis, and ACLF. Plasma levels of ARA and DHA were similar in patients with compensated cirrhosis, decompensated cirrhosis, and ACLF. Furthermore, no significant association between plasma ARA or DHA and C-reactive protein or peripheral blood leukocytes were observed (P>0.05).

**Conclusion:**

In our study plasma levels of key omega-3 and omega-6 fatty acid are neither associated with the severity of liver cirrhosis nor with liver-cirrhosis-associated systemic inflammation.

## Introduction

Polyunsaturated fatty acids (PUFA) are important for the structure and function of cell membranes and have additional important functions in regulating immune and inflammatory responses as precursors of eicosanoids, resolvins and other lipid mediators[1]. Eicosanoids (for example prostaglandins and leukotrienes) which are derived from arachidonic acid (ARA) and associated omega-6 PUFAs exhibit pro-inflammatory and pro-coagulatory functions. In contrast, eicosanoids, resolvins and other mediators derived from omega-3 fatty acids such as docosahexaenoic acid (DHA) and eicosapentaenoic acid (EPA) support the resolution of inflammation [1,2]. Hence, the balance between proinflammatory and proresolving eicosanoids appears to be of pivotal importance for the regulation and appropriate termination of inflammatory response [2,3]. The ratio of omega-6/omega-3 PUFAs is an important health determinant and a shift in the balance towards omega-3 PUFAs leads to a lower death rate in cardiovascular disease [4–6]. A role for omega-3 PUFAs in ameliorating rheumatoid arthritis, asthma as well as diabetes and cancer has also been described[7] [8–10]. In liver disease, omega-3 PUFAs may reduce hepatic lipogenesis, inflammation and hepatic fibrosis[11–13]. A recent meta-analysis conducted by *Yan et al*. suggests that omega-3 PUFA supplementation may decrease liver fat and hepatic enzyme parameters in patients with non-alcoholic fatty liver disease[14].

Decompensated liver cirrhosis is associated with a profound chronic systemic inflammatory response, which can exacerbate during the development of acute-on-chronic liver failure (ACLF) and contribute to the pathogenesis of ACLF-defining organ failures [15,16]. Of note, cirrhosis-associated systemic inflammation is paralleled by a state of profound immunosuppression[16–18]. Hence, it can be speculated that an inappropriate resolution of inflammation contributes to the pathogenesis of complications of liver cirrhosis.

Patients with liver cirrhosis have a lack of crucial fatty acids including PUFAs like ARA and DHA, which could be explained by a reduced alimentary intake as well as an impaired synthesis in the liver and an increased degradation of PUFA due to lipid peroxidation [4–6,19]. For example *Arain et al*. observed that patients with HBV-cirrhosis have an altered total fatty acid composition in comparison to controls with significantly reduced levels of ARA and EPA in cirrhotic patients[20]. *Basili et al*. showed that increased ratios of omega-3/omega-6 PUFAs are related to disease severity as well as higher oxidative stress in cirrhotic patients[21]. Furthermore *Obrien et al* found elevated prostaglandin (PGE2) levels, a typical eicosanoid derived from ARA, in acutely decompensated patients at immunosuppressive levels[22]. However, little is known about the relevance of omega-3 and -6 fatty acids to regulate cirrhosis-associated immune dysfunction during the development of ACLF. In the present study we therefore aimed to explore the relationship between plasma levels of key omega-3 and -6 fatty acids and liver cirrhosis-associated inflammation and development of ACLF.

## Patients and methods

### Study population

Consecutive patients admitted to the University Hospital Frankfurt, Germany, with acute decompensation of liver cirrhosis and / or acute-on-chronic liver failure according to the criteria of the CLIF-EASL consortium [15], were prospectively enrolled in our liver cirrhosis cohort study since August 2013. In 2015, the cohort was extended to patients with compensated liver cirrhosis. The diagnosis of liver cirrhosis was based by combination of clinical, laboratory and imaging findings (ultrasound and transient elastography or share wave elastography) or–rarely—by liver biopsy. Acute decompensation of liver cirrhosis was defined as presence of one of the following criteria: new onset / progression of hepatic encephalopathy graded by West-Haven criteria [23], gastrointestinal hemorrhage, bacterial infection, or ascites grade II-III (graded according to *Moore et al*.[24]). ACLF was diagnosed according to the ACLF-criteria proposed by the CLIF-EASL consortium [15]. Clinical data and biomaterials of patients with acute decompensation of liver cirrhosis or ACLF were collected at baseline and during follow-up days 7 and 28 as well as follow-up week 12. Clinical data and biomaterials of patients with compensated or stable decompensation of liver cirrhosis were collected at baseline and at least every 3 months of follow-up, or at the time of development of acute decompensation or ACLF.

In the present post-hoc subanalysis of our cohort study, all patients were included who were recruited until April 2017 and of whom plasma samples were available. Exclusion criteria were age below 18 years, pregnancy or breastfeeding, presence of hepatocellular carcinoma (HCC) beyond Milan criteria, presence of infection with human immunodeficiency virus (HIV), or therapy with immunosuppressive agents.

All patients provided written informed consent to the study protocol, and the study was approved by the local ethic committee of the University Hospital Frankfurt, Germany.

### Determination of docosahexaenoic acid and arachidonic acid concentrations

The concentrations of docosahexaenoic acid (DHA) and arachidonic acid (ARA) in serum samples were determined by liquid chromatography-electrospray ionization-tandem mass spectrometry (LC-ESI-MS/MS).

A standard solution of ARA (50 μg/mL) and of DHA (500 ng/mL, both obtained from Cayman Chemical, Ann Arbor, USA) was prepared in methanol. This solution was further diluted with methanol/butylhydroxytoluol (BHT) (100:0.1 v/v) to obtain working standard solutions covering the following concentration ranges: 2.5 ng/mL to 500 ng/mL for ARA and 0.025 ng/mL to 5 ng/mL for DHA.

Calibration standard samples and quality control (QC) samples were prepared by spiking 50 μL PBS with 20 μL standard working solution and 20 μL internal standard solution ([^2^H_8_]- ARA (125 ng/mL) and [^2^H_5_]-DHA (10 ng/mL both obtained from Cayman Chemical, Ann Arbor, USA)). Serum samples were diluted by factor 5 and 10 and spiked with 20 μL methanol/BHT (100:0,1 v/v) and 20 μL internal standard solution.

Afterwards, analytes were extracted from the prepared standard, QC and serum samples by liquid-liquid extraction with ethyl acetate. 600 μL of ethyl acetate were added to the prepared sample, the mixture was vortexed and centrifuged at 20,000 g for 5 minutes. The organic phase was transferred to a new tube. The extraction step was repeated and the combined organic phases were evaporated at a temperature of 45°C under a gentle stream of nitrogen. The residues were reconstituted with 50 μL of methanol/water/BHT (50:50:10–4, v/v/v), centrifuged for 2 minutes at 10,000 g and then transferred to glass vials prior to injection into the LC-MS/MS system.

DHA and ARA were analyzed using a Gemini NX column (150 x 2 mm I.D., 5 μm particle size and 110 Å pore size from Phenomenex, Aschaffenburg, Germany) coupled to a hybrid triple quadrupole tandem mass spectrometer 5500QTRAP (Sciex, Darmstadt, Germany). Separation was carried through under gradient conditions with solvent A (0.01% ammonia in water) and solvent B (0.01% ammonia in acetonitrile). The following gradient was employed: 0 min 85% A, 4 min 60% A, 6–7 min 10% A, 7.5–11.5 min 85% A. MS parameters were set as follows: Ionspray voltage -4500 V, source temperature 550°C, curtain gas 35 psi nebulizer gas 50 psi, heating gas 50 psi, collision gas 9 psi. The analysis was done in multiple reaction monitoring (MRM) mode with all quadrupoles running at unit resolution. The following precursor-to-product ion transitions were used for quantification: m/z 327.3 → 229.0 for DHA (declustering potential -140 V, collision energy -17 V) and m/z 303.0 → 40.9 for ARA (declustering potential -175 V, collision energy -80 V).

Data acquisition was done using Analyst Software V.1.6.2, quantitation was performed with MultiQuant Software V3.02. (Sciex, Darmstadt, Germany) using the internal standard method (isotope-dilution mass spectrometry). Ratios of analyte peak area and internal standard peak area (y-axis) were plotted against concentration (x-axis) and calibration curves for each analyte were calculated by linear regression with 1/concentration weighting.

### Statistical analyses

Statistical analyses were performed using BiAS, Version 11.06, and Graphpad PRISM5. Group differences were assessed by means of χ^2^ contingency tables or Wilcoxon-Mann-Whitney-U-tests, as appropriate. *P* values < 0.05 were considered to be statistically significant. Associations of outcomes with continuous or dichotomic variables were assessed in linear and logistic regression models, respectively. After univariate analyses, multivariate analyses were performed for significant associations. Multivariate models were obtained by backward selection, using a *P* value >0.15 for removal from the model.

## Results

### Study population–baseline characteristics

A total of 117 patients were included in the present analyses according to the above defined selection criteria. Of those, 26 (22.2%), 51 (43.6%), and 40 (34.2%) patients had compensated liver cirrhosis, decompensated liver cirrhosis, and ACLF at baseline, respectively. Overall, 22 patients died until follow-up week 12. As described previously, patients with ACLF had higher levels of systemic inflammation than patients with decompensated liver cirrhosis (Table 1). Baseline characteristics of included patients are shown in Table 1.

**Table 1:** Baseline characteristics and laboratory results of included patients.

	ACLF (n = 40)	decompensated (n = 51)	compensated (n = 26)	P-value (ACLF vs. decompensated)	P-value (decompensated vs. compensated)
Age (years), mean (SD)	55 (10)	55 (11)	55 (12)	0.7	1.0
Male gender, n (%)	30 (75.0)	36 (70.5)	17 (65.4)	0.8	0.8
BMI (kg/m^2^), mean (SD)	27.8 (6.8)	25.2 (5.7)	26.4 (5.9)	0.2	0.6
**Origin of cirrhosis**					
Alcohol, n (%)	23 (58)	30 (59)	13 (50)	1.0	0.8
HCV/HBV, n (%)	4 (10)	4 (8)	5 (19)	1.0	0.4
NASH, n (%)	2 (5)	3 (9)	1 (4)	1.0	1.0
Other, n (%)	11 (28)	14 (27)	7 (27)	1.0	1.0
**Laboratory data**					
Leucocytes (/nl), mean (SD)	11.97 (6.67)	8.47 (4.88)	6.1 (5.1)	**0.01**	**0.003**
Hemoglobin (g/dl), mean (SD)	9.3 (1.7)	10.7 (2.8)	11.1 (2.3)	**0.008**	0.6
Platelets (/nl), mean (SD)	108 (72)	135 (97)	107 (61)	0.1	0.3
CRP (mg/dl), mean (SD)	5.4 (4.6)	2.7 (2.9)	1.5 (2.1)	**<0.001**	**0.07**
Sodium (mmol/l), mean (SD)	129 (20)	135 (6)	136 (4)	**0.06**	0.4
Creatinine (mg/dl), mean (SD)	2.7 (1.4)	1.0 (0.4)	0.90 (0.30)	**<0.001**	0.2
Bilirubin (mg/dl), mean (SD)	15.1 (13.7)	7.1 (9.3)	4.6 (5.7)	**0.01**	0.2
ALT (U/l), mean (SD)	51 (40)	53 (45)	36 (26)	0.8	0.09
γGT (U/l), mean (SD)	191 (187)	164 (183)	146 (144)	0.3	1.0
INR, mean (SD)	2.16 (0.86)	1.53 (0.45)	1.45 (0.35)	**<0.001**	**0.6**
Albumin (g/dl), mean (SD)	2.9 (0.6)	2.8 (0.5)	3.2 (0.7)	0.9	0.1
Docosahexaenoic acid (ng/ml), mean (SD)	17 (11)	18 (14)	18 (19)	0.8	0.6
Arachidonic acid (ng/ml), mean (SD)	2060 (1035)	2524 (2735)	1972 (929)	0.8	0.9
ARA/DHA, mean (SD)	147 (77)	161 (70)	166 (78)	0.2	0.6

ALT, alanine aminotransferase; ARA, arachidonic acid; AST, aspartate aminotransferase; BMI, body mass index; CRP, C-reactive protein; DHA, docosahexaenoic acid; γGT, γ-glutamyl transferase; HBV, hepatitis B virus; HCV, hepatitis C virus; INR, international normalized ratio; NASH, nonalcoholic steatohepatitis; SD, standard deviation

### No association between arachidonic acid and docosahexaenoic acid plasma levels and severity of liver cirrhosis

**Fig 1:**
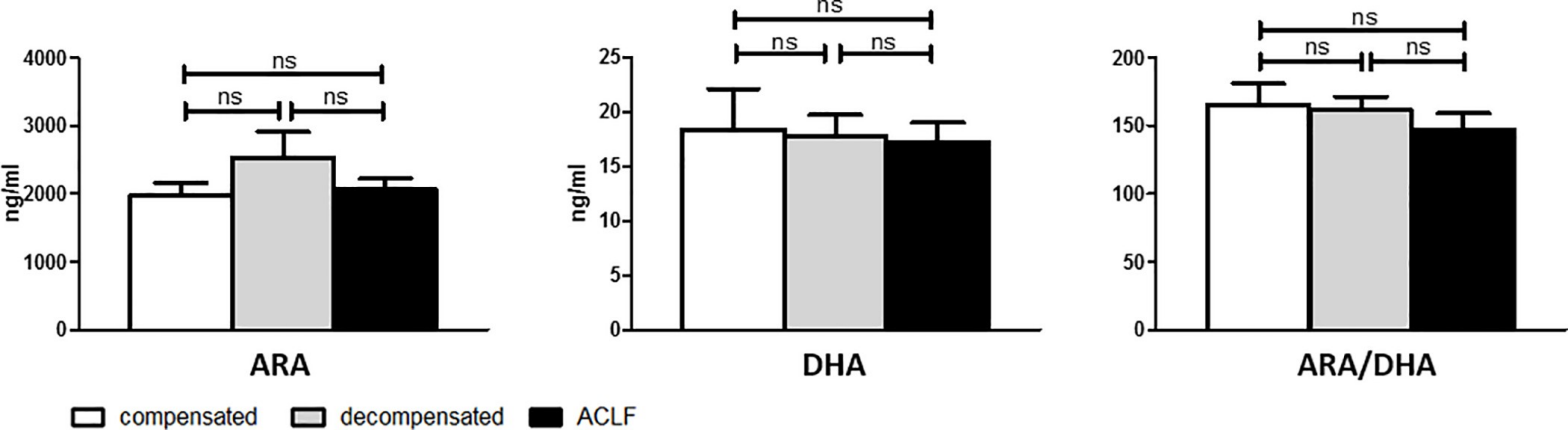
Plasma concentrations of omega-3 and -6 fatty acids according to the severity of liver cirrhosis. ARA, arachidonic acid. DHA, docosahexaenoic acid.

**Fig 2:**
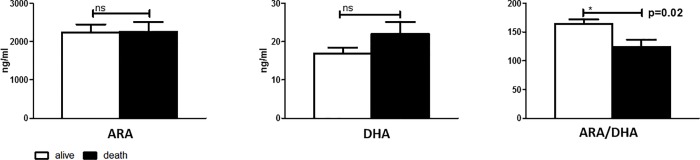
Plasma concentrations of omega-3 and -6 fatty acids in patients who died or survived until week 12 of follow-up. ARA, arachidonic acid. DHA, docosahexaenoic acid.

As important representatives of omega-6 and omega-3 PUFA precursors of pro- and anti-inflammatory lipid mediators, ARA and DHA levels were quantified in baseline plasma of all included patients. Plasma levels of ARA and DHA were similar in patients with compensated cirrhosis, decompensated cirrhosis and ACLF (Fig 1). There was also no significant difference in the ratios of ARA to DHA according to the severity of liver disease (compensated vs. decompensated cirrhosis vs. ACLF: 166 vs. 161 vs. 147, not significant). In addition, there was no difference in baseline plasma levels of ARA and DHA in patients who died or who survived during follow-up (Fig 2), although the ratio of ARA to DHA was slightly lower in patients who died in comparison to those who survived (P = 0.02).

### Arachidonic acid and docosahexaenoic acid plasma levels are not associated with systemic inflammation in patients with liver cirrhosis

**Fig 3:**
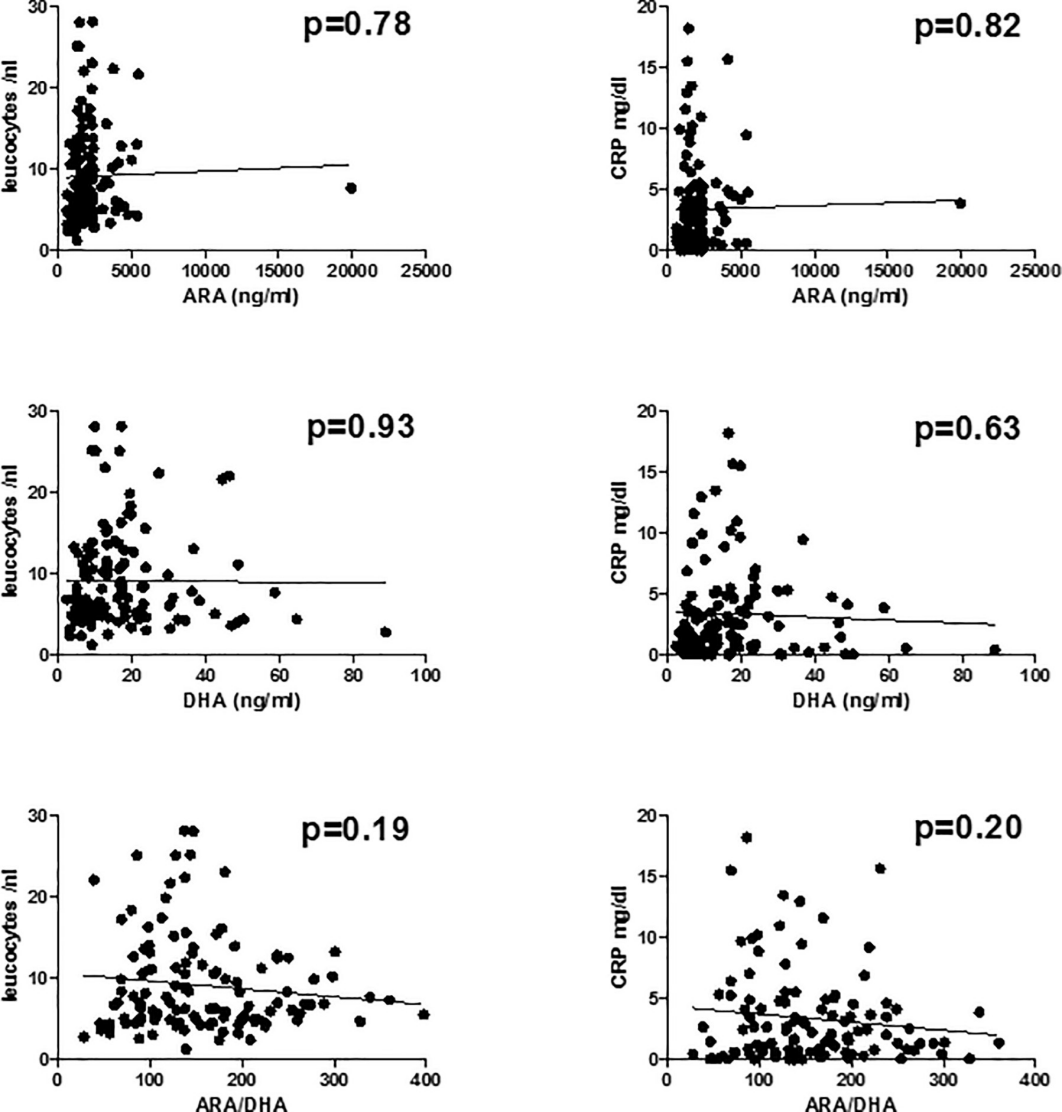
Association between plasma omega-3 and -6 fatty acids and cirrhosis-associated systemic inflammation. ARA, arachidonic acid. CRP, C-reactive protein. DHA, docosahexaenoic acid.

**Fig 4:**
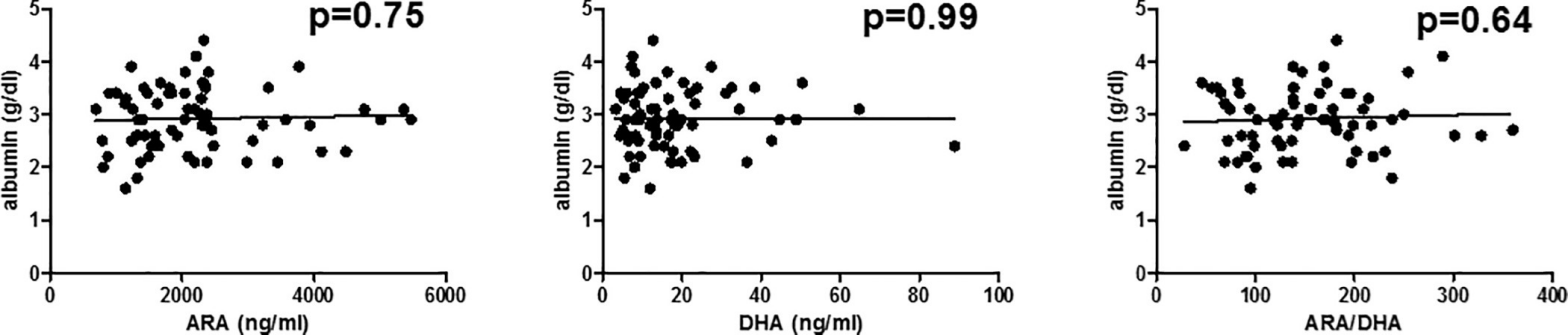
Association between plasma omega-3 and -6 fatty acids and serum albumin. ARA, arachidonic acid. DHA, docosahexaenoic acid.

We next tested possible associations between ARA, DHA and established markers of liver cirrhosis-associated systemic inflammation. There was no significant association between plasma ARA, DHA, or the ratio between ARA/DHA, and C-reactive protein or peripheral blood leukocytes in our cohort (P>0.15 for each correlation, Fig 3). Finally, we correlated plasma ARA, DHA, and ARA/DHA-ratio with serum albumin levels, as albumin has important immunomodulatory functions in patients with liver cirrhosis [25]. Again, no correlation between concentrations of albumin and ARA, DHA or the ratio of ARA/DHA was observed in our cohort, Fig 4.

## Discussion

In the present post-hoc-analysis of a prospective cohort study of patients with compensated liver cirrhosis, decompensated liver cirrhosis, or ACLF, plasma levels of ARA and DHA and their ratio are neither associated with the severity of liver disease/survival, nor with the magnitude of liver cirrhosis-associated systemic inflammation. This finding is in contrast to the well-known increase of eicosanoids, i.e. the downstream metabolites of ARA/DHA, in patients with progressive liver disease.

Previous studies have shown decreased levels of ARA and DHA in patients with liver cirrhosis compared to healthy controls [20–22]. Yet, *Obrien et al*. [22] found that plasma Prostaglandin E2 (PGE_2_) levels, a classical eicosanoid and downstream product of ARA, were sevenfold higher in patients with acutely decompensated liver than in healthy subjects. An increased synthesis of prostaglandins appeared to be associated with decreased bacterial killing and contribute to immune paralysis of liver cirrhosis, which could be partially reverted by supplementation of human serum albumin.

*Basili et al*.[21] showed that cirrhotic patients in comparison to healthy controls had a higher ratio of omega-6/omega-3 PUFAs, which correlated with disease severity and oxidative stress markers. However most of the 51 included patients had CHILD A cirrhosis (31) and only 3 patients were classified as CHILD C cirrhosis. Furthermore patients presented in an outpatient clinic or were admitted to hospital for elective procedures. In summary they analyzed a cohort consisting of stable cirrhotic patients whereas our cohort consisted of patients covering the whole spectrum from compensated liver cirrhosis to ACLF and death from ACLF.

Collectively, our data suggest that the increase of pro- and anti-inflammatory eicosanoids in patients with advanced liver cirrhosis does not correlate with plasma levels (and therefore–likely–not with nutritional uptake) of their omega-3 and -6 PUFA precursors ARA and DHA. Instead, the induction / repression of enzymes generating pro- and anti-inflammatory PUFA-derived lipid mediators may be the predominant mechanism of their increased production in advanced liver cirrhosis.

The high ratio of omega-6/omega-3 PUFAs and the altered profile of fatty acids in patients with liver cirrhosis, which may be explained by a reduced alimentary intake as well as an impaired synthesis in the liver and an increased degradation of PUFAs due to lipid peroxidation[4–6] may lead to higher oxidative stress[21] as well as hepatic fibrosis and inflammation [11–13]. Yet, our data suggest that the serum concentrations of omega-6/omega-3 PUFAs do not play a relevant role in the phase of acute decompensation of liver cirrhosis with severe deterioration of liver function, systemic inflammation and development of organ failures. In contrast, our finding, that neither ARA, DHA, nor their ratio in plasma are associated with liver cirrhosis-associated systemic inflammation may suggest that omega-3 PUFA-derived pro-resolution mediators such as resolvins may not be sufficient to promote the resolution of liver cirrhosis-associated inflammation. Indeed, we have failed to detect lipoxins and resolvins in the plasma of our patients (not shown). This observation is in line with a previous study showing that supplementation of high doses of omega-3 fatty acids does not result in detectable levels of these key pro-resolution mediators [26]. These and our data may suggest a limited role of endogenous pro-resolution mediators in the resolution of inappropriate inflammatory responses. Alternative strategies such as administration of pharmacological doses of pro-resolution mediators themselves might be necessary to ameliorate liver cirrhosis-associated inflammation [2], although our data are not sufficient to proof this assumption.

Our study has a number of limitation. First of all, our findings are limited by the exclusive characterization of ARA / DHA in plasma, whereas tissue concentrations of these PUFAs might show a different pattern. Additionally, we only studied patients with advanced liver disease (compensated cirrhosis up to ACLF) and did not determine ARA and DHA in a healthy control cohort. Another limitation is the lacking information of nutritional uptake of PUFAs in our study.

In conclusion, plasma levels of ARA / DHA are neither associated with systemic inflammation nor with the severity of liver cirrhosis.
